# Supplementary material for: Microbiota-assisted iron uptake promotes immune tolerance in the intestine
Source: Nat Commun. 2023 May 15;14:2790. doi: 10.1038/s41467-023-38444-2 (PMC10185671; doi:10.1038/s41467-023-38444-2)
Supplement: Supplementary file 1 — Supplementary Information [file 41467_2023_38444_MOESM1_ESM.pdf]

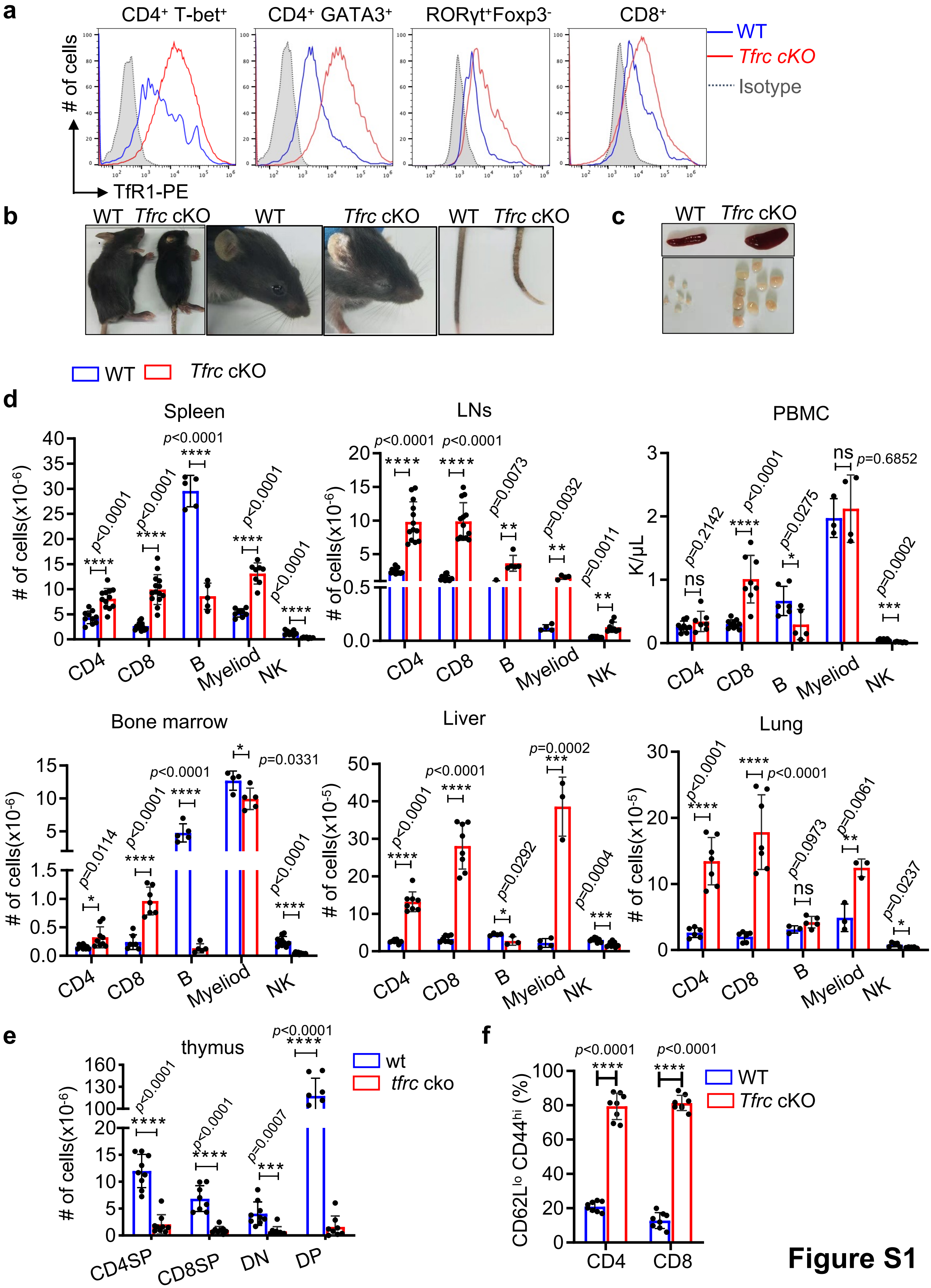
a
CD4⁺ T-bet⁺
CD4⁺ GATA3⁺
RORγt⁺Foxp3⁻
CD8⁺
WT
Tfrc cKO
Isotype
# of cells
TfR1-PE
b
WT Tfrc cKO
WT
Tfrc cKO
WT Tfrc cKO
c
WT Tfrc cKO
WT Tfrc cKO
d
Spleen
LNs
PBMC
# of cells(x10-6)
K/μL
Bone marrow
Liver
Lung
# of cells(x10-5)
CD4
CD8
B
Myeliod
NK
e
thymus
wt
tfrc cko
CD4SP
CD8SP
DN
DP
f
CD62L lo CD44hi (%)
WT
Tfrc cKO
CD4
CD8

Figure S1

Supplementary Figure Legends

Figure S1. Phenotypic analysis of 21-day-old *Tfrc* cKO mice

(**a**). TfR1 was specifically abolished in Treg cells of *Tfrc* cKO mice. As in **Figure 1a**, expression of TfR1 in splenic Th1 cells ($CD4^{+}Tbet^{+}$), Th2 cells ($CD4^{+}Gata\text{-}3^{+}$), Th17 cells ($CD4^{+}Foxp3^{-}ROR\gamma t^{+}$), and CD8 T cells were determined by flow cytometry. Grey-shaded areas represent staining with an isotype control antibody. The data are representative of 3 independent experiments.

(**b**). Representative images of 21-day-old *Tfrc* cKO mice with smaller body sizes, dermatitis, and squinted eyes. WT littermates were included as negative controls.

(**c**). Representative images of the enlarged spleens and lymph nodes of 21-day-old *Tfrc* cKO mice.

(**d**). Numbers of indicated immune cells were determined by flow cytometry in the indicated organs of 3-week-old *Tfrc* cKO mice. Each dot represents one individual mouse from 4 independent experiments.

(**e**). CD4 SP (CD4+CD8-), CD8 SP (CD4-CD8+), DP (CD4+CD8+), and DN (CD4-CD8-) cells in the thymus were determined by flow cytometry. The data are representative of five independent experiments (CD4 SP, n=9 mice per group; CD8 SP, n=8 mice per group; DN, n=9 mice per group; DP, n=8 mice per group).

(**f**). Summary of effector T cell ($CD44^{hi}CD62L^{lo}$) percentages in the spleen of 21-day-old *Tfrc* cKO and WT littermates. The data are representative of at least three independent experiments (n= 8 mice per group).

(**d,e,f**), Statistical significance was determined by two-tailed Student's t test, data are mean ± SD. *$p<0.05$, **$P<0.01$, ***$p<0.001$, **** $p<0.0001$.

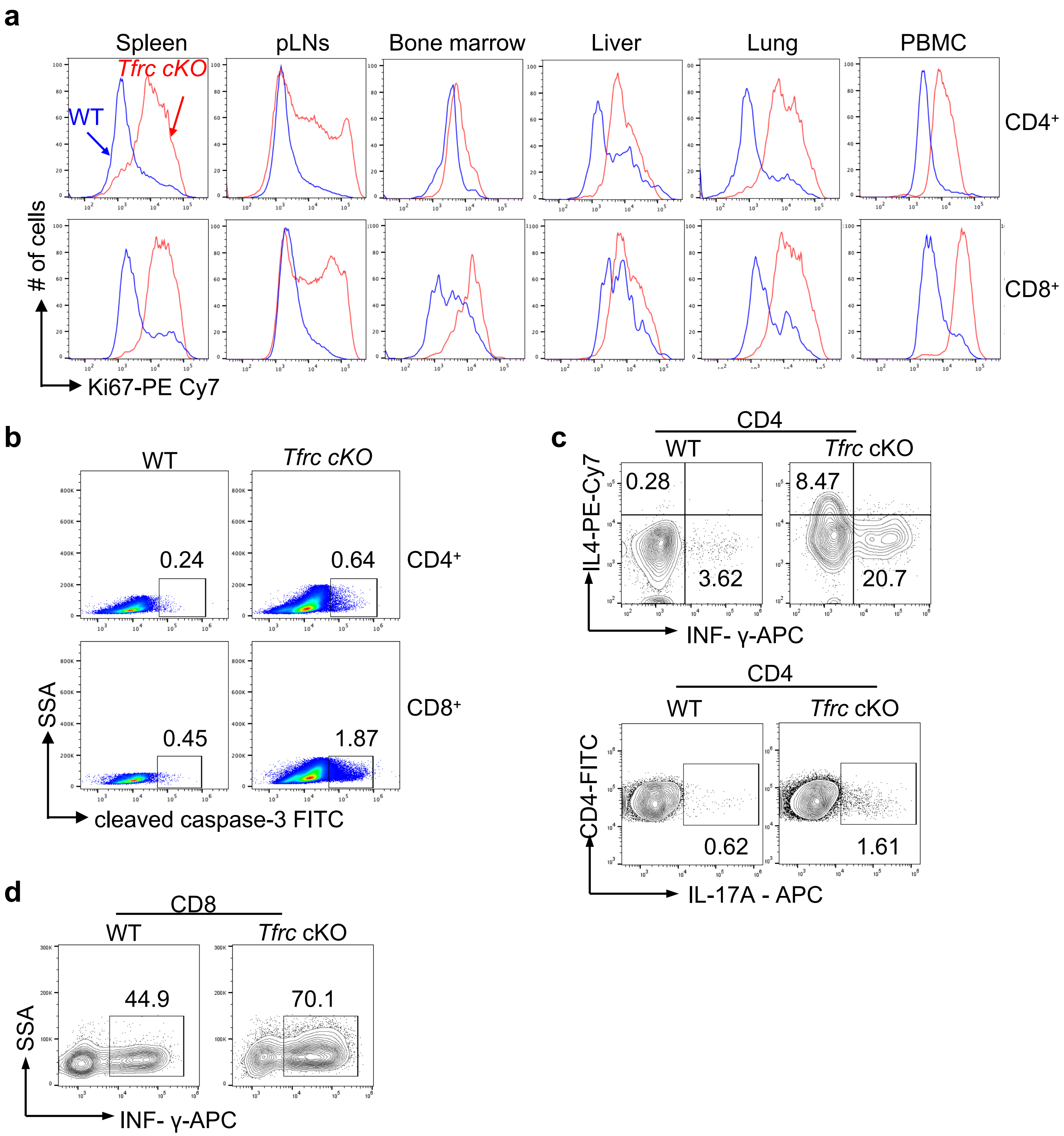
a
Spleen
pLNs
Bone marrow
Liver
Lung
PBMC
Tfrc cKO
WT
CD4⁺
CD8⁺
# of cells
Ki67-PE Cy7
b
WT
Tfrc cKO
0.24
0.64
CD4⁺
0.45
1.87
CD8⁺
SSA
cleaved caspase-3 FITC
c
CD4
WT
Tfrc cKO
0.28
3.62
8.47
20.7
IL4-PE-Cy7
INF- γ-APC
CD4
WT
Tfrc cKO
0.62
1.61
CD4-FITC
IL-17A - APC
d
CD8
WT
Tfrc cKO
44.9
70.1
SSA
INF- γ-APC

Figure S2

Figure S2. Increased proliferation of T cells contributes to their accumulation in 21-day-old *Tfrc* cKO mice.

(**a**) Cellular proliferation was determined by Ki-67 staining of CD4 (upper panels) and CD8 T cells (lower panels) from indicated organs. Data are representative of four independent experiments.

(**b**). Cell death was determined by cleaved Caspase 3 staining of CD4 and CD8 T cells in the spleen. Data are representative of three independent experiments.

(**c**). Representative FACS staining of IFNγ-, IL-4- and IL-17A-expressing CD4 T cells in the spleens of 3-week-old *Tfrc* *c*KO and WT mice. Splenocytes were stimulated with PMA and ionomycin for 3 hours in the presence of Golgi-stop. IL-17A-, IL-4- and IFNγ-expressing CD4 T cells were determined by intracellular staining.

(**d**). Representative FACS staining of IFNγ^{+} cells from 21-day-old *Tfrc c*KO and WT CD8 T cells. The IFNγ expression in CD8 T cells was determined by intracellular staining following PMA/ionomycin stimulation.

(**c-d**). The data are summary or representative of at least three independent experiments.

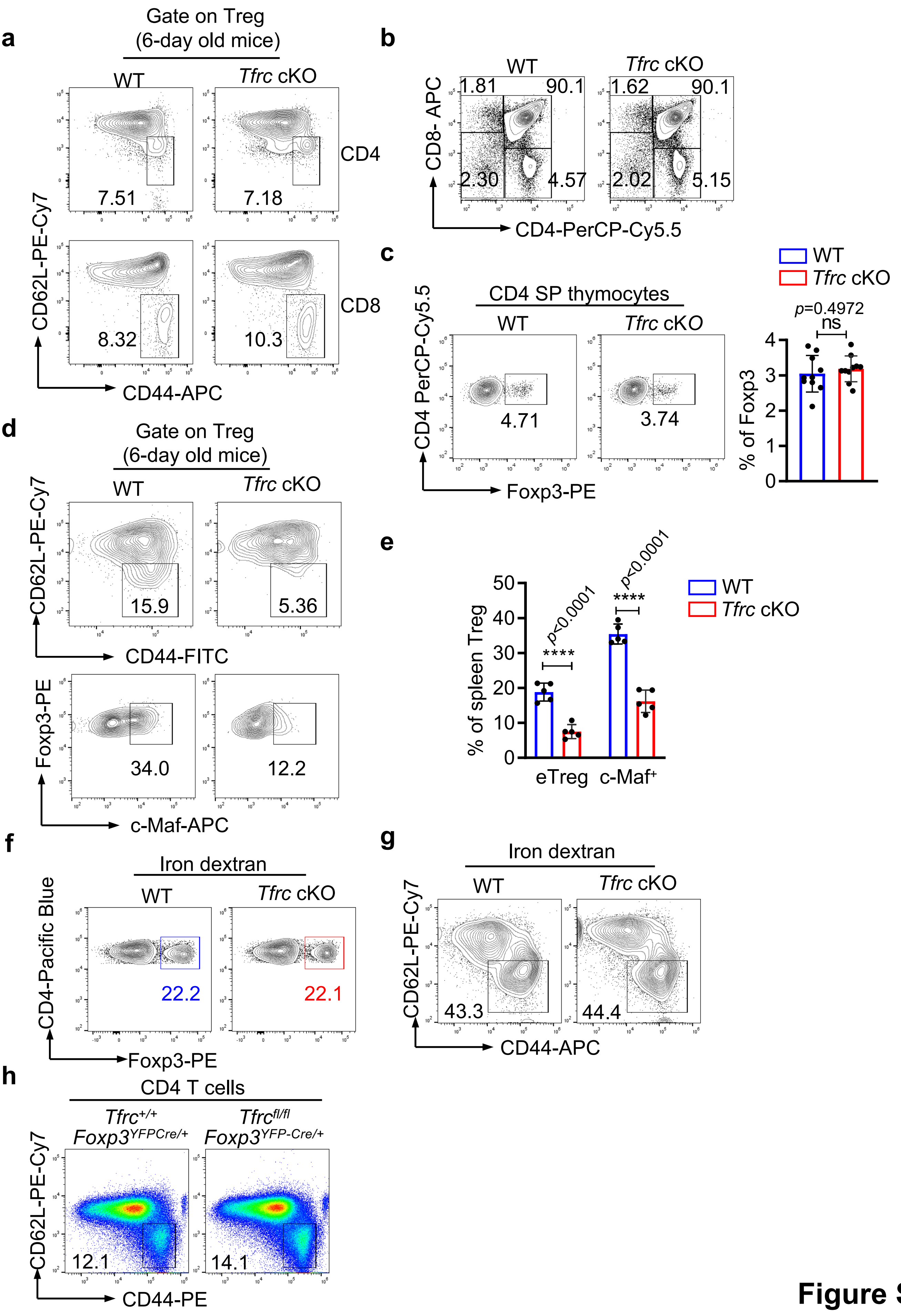

a
Gate on Treg
(6-day old mice)
WT
Tfrc cKO
CD4
CD8
7.51
7.18
8.32
10.3
CD62L-PE-Cy7
CD44-APC
b
WT
Tfrc cKO
CD8- APC
CD4-PerCP-Cy5.5
1.81
90.1
2.30
4.57
1.62
90.1
2.02
5.15
c
CD4 SP thymocytes
WT
Tfrc cKO
CD4 PerCP-Cy5.5
Foxp3-PE
4.71
3.74
WT
Tfrc cKO
p=0.4972
ns
% of Foxp3
d
Gate on Treg
(6-day old mice)
WT
Tfrc cKO
CD62L-PE-Cy7
CD44-FITC
15.9
5.36
Foxp3-PE
c-Maf-APC
34.0
12.2
e
p<0.0001
p<0.0001
****
****
% of spleen Treg
eTreg
c-Maf+
WT
Tfrc cKO
f
Iron dextran
WT
Tfrc cKO
CD4-Pacific Blue
Foxp3-PE
22.2
22.1
g
Iron dextran
WT
Tfrc cKO
CD62L-PE-Cy7
CD44-APC
43.3
44.4
h
CD4 T cells
Tfrc+/+ Foxp3YFPCre/+
Tfrcfl/fl Foxp3YFP-Cre/+
CD62L-PE-Cy7
CD44-PE
12.1
14.1

Figure S3

Figure S3. Phenotypic analysis of 6-day-old *Tfrc* cKO mice.

(**a**). Comparing the activation statuses of T cells in the spleens of 6-day-old *Tfrc* cKO mice. $CD4^+$ and $CD8^+$ T cells in the spleens of 6-day-old *Tfrc* cKO mice and their WT littermates were analyzed for the expression of CD44 and CD62L. Effector/memory T cells were gated as $CD62L^{low}CD44^{hi}$. The data are representative of three independent experiments.

(**b, c**). Total thymocytes isolated from 10-day-old WT and *Tfrc* cKO mice were analyzed for the expression of CD4 and CD8 (**b**). Foxp3 expression in the gated CD4 SP cells was further analyzed by intracellular staining (**c**). n = 10 mice per group from three independent experiments.

(**d,e**). The numbers of eTreg and $c\text{-}Maf^+$ Treg cells were decreased in 6-day-old *Tfrc* cKO mice. $CD4^+Foxp3^+$ cells in the spleen were analyzed for the expression of CD44/CD62 L (upper panels) and c-Maf (lower panels). Effector Treg cells were gated as $Foxp3^+CD62L^{low}CD44^{hi}$. (**d**). Representative flow cytometry analysis; (**e**). Summary of the percentages of effector Treg cells and $c\text{-}Maf^+$ Treg cells in splenic Treg cells from three independent experiments (n=5 mice per group).

(f,g). Neonatal (3-day-old) *Tfrc* cKO mice and WT littermates were injected intraperitoneally with iron dextran on postnatal day 3 (5 mg e.a.), day 9 (7.5 mg e.a.), and every 7 days (12.5 mg/ e.a.) thereafter until reaching 6 weeks of age. (**f),** Representative FACS staining showing the ratios of Tregs in CD4 T cells from 6-week-old *Tfrc c*KO and WT mice receiving the iron dextran. (**g**), The expression of CD44 and CD62L in $CD4^+$ splenocytes from iron dextran-treated WT and *Tfrc c*KO mice was analyzed. The data are representative of three independent experiments.

(**h**) Flow cytometry analysis of the CD44 and CD62L expression in $CD4^+$ splenocytes from $Tfrc^{fl/fl}$ $Foxp3^{YFP\text{-}Cre/+}$ and $Tfrc^{+/+}$ $Foxp3^{YFP\text{-}Cre/+}$ female mice. The data are representative of three independent experiments.

(**c,e**) Statistical significance was determined by a two-tailed *t test*. Data are mean ± SD. ****, $P<0.0001$ by two-tailed Student's *t* test.

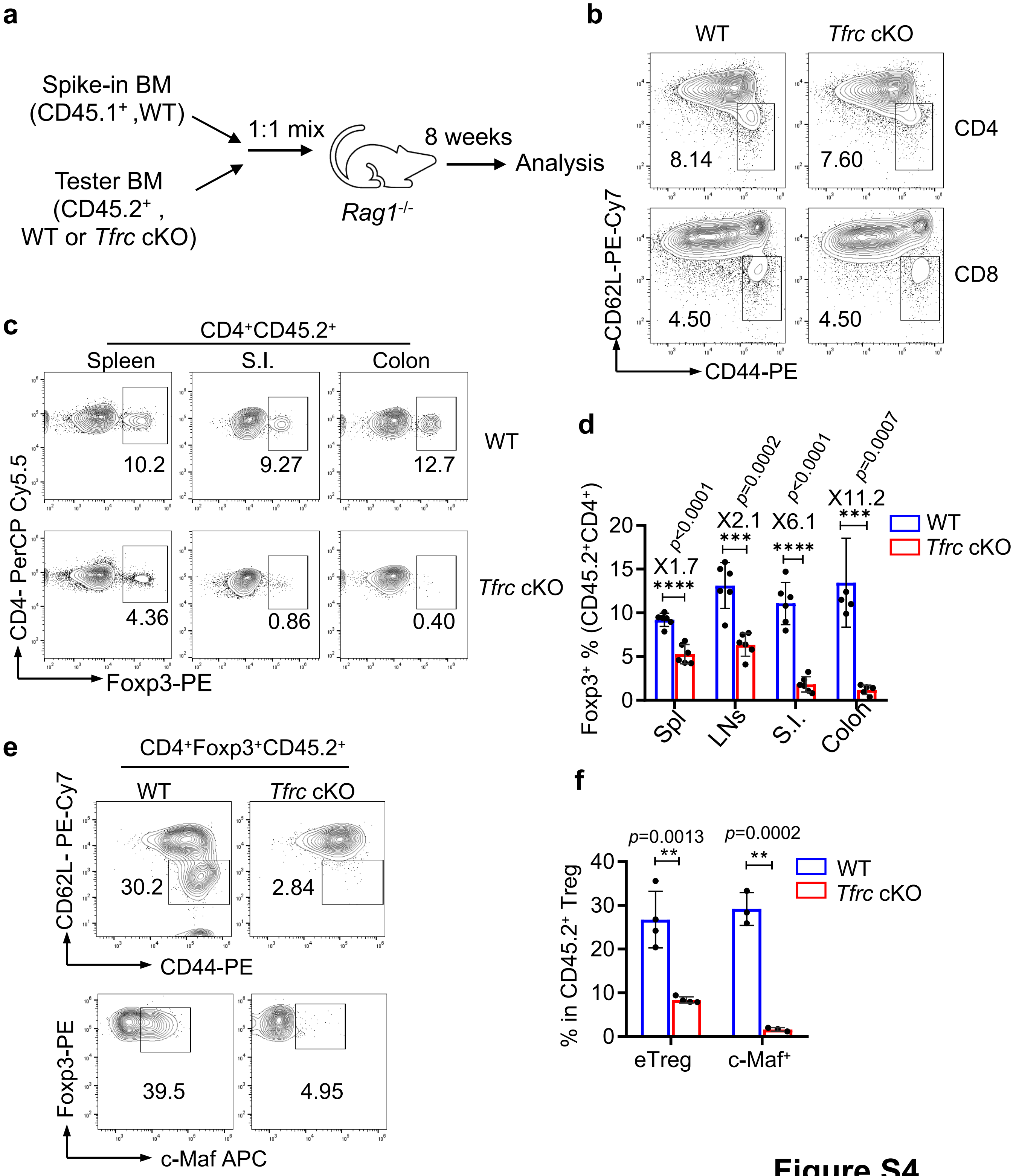
a
Spike-in BM (CD45.1+ ,WT)
Tester BM (CD45.2+ , WT or Tfrc cKO)
1:1 mix
Rag1-/-
8 weeks
Analysis
b
WT
Tfrc cKO
CD4
CD8
8.14
7.60
4.50
4.50
CD62L-PE-Cy7
CD44-PE
c
CD4+CD45.2+
Spleen
S.I.
Colon
WT
Tfrc cKO
10.2
9.27
12.7
4.36
0.86
0.40
CD4- PerCP Cy5.5
Foxp3-PE
d
Foxp3+ % (CD45.2+CD4+)
p<0.0001
p=0.0002
p<0.0001
p=0.0007
X1.7
X2.1
X6.1
X11.2
****
***
****
***
WT
Tfrc cKO
Spl
LNs
S.I.
Colon
e
CD4+Foxp3+CD45.2+
WT
Tfrc cKO
30.2
2.84
CD62L- PE-Cy7
CD44-PE
39.5
4.95
Foxp3-PE
c-Maf APC
f
% in CD45.2+ Treg
p=0.0013
p=0.0002
**
**
WT
Tfrc cKO
eTreg
c-Maf+

Figure S4

Figure S4. Bone marrow chimeric experiments revealed a cell-intrinsic function of TfR1 in the differentiation of c-Maf^{+} Treg cells.

(**a**) Schematic of the bone marrow chimeric mice. *Rag1*$^{-/-}$ mice were reconstituted with *Tfrc c*KO (*Tfrc*$^{fl/fl}$ *Foxp3*$^{YFP\text{-}IRES\text{-}Cre/y}$ mice, CD45.2^{+}) bone marrow cells mixed with B6.SJL (CD45.1^{+}) bone marrow cells or WT mouse (*Tfrc*$^{+/+}$*Foxp3*$^{YFP\text{-}IRES\text{-}Cre/y}$ mice CD45.2^{+}) bone marrow cells mixed with B6.SJL (CD45.1^{+}) bone marrow cells as indicated. The recipient mice were analyzed 8 weeks after reconstitution.

(**b**). Spontaneous T-cell activation was lacking in the chimeric mice. Total CD4 (upper panels) and CD8 (lower panels) T cells from the spleens of the chimeric mice were analyzed for the expression of CD44 and CD62L. Effector/memory cells were gated as CD44^{hi}CD62L^{low}.

(**c,d**). *Tfrc* cKO bone marrow-derived Treg cells were completely lost in the intestines of chimeric mice. CD4^{+} T cells from the spleens, small intestines (S.I.), and colons of the chimeric mice were first separated based on the expression of CD45.1 (B6. SJL) or CD45.2 (*Tfrc* cKO or control WT), and Foxp3 expression was determined in CD4^{+}CD45.2^{+} T cells by intracellular staining. (**c**). Representative flow cytometry analysis; (**d**). Summary of the percentage of Foxp3^{+} in CD4^{+}CD45.2^{+} cells (n=6 mice per group in the spleen, LNs and S.I.; n=5 mice per group in the colon) based on two independent experiments.

(**e,f**). Diminished eTreg and c-Maf^{+} Treg cells derived from *Tfrc* cKO bone marrow. CD4^{+}CD45.2^{+}Foxp3^{+} cells in the spleens of the chimeric mice were gated and further analyzed for CD44/CD62L (upper panels) and c-Maf (lower panels) expression by flow cytometry. (**e**). Representative flow cytometry analysis; (**f**). Summary of the percentages of eTreg (CD62L^{low}CD44^{hi}) or c-Maf^{+} cells among the gated CD4^{+}CD45.2^{+}Foxp3^{+} cells (for eTreg, n=4 mice per group; for c-Maf^{+} Treg, n=3 mice per group based on 2 independent experiments).

The error bars represent the standard deviation of the mean, **, p<0.01, ****p*<0.001, *****p*<0.0001 in two-tailed Student's *t test*.

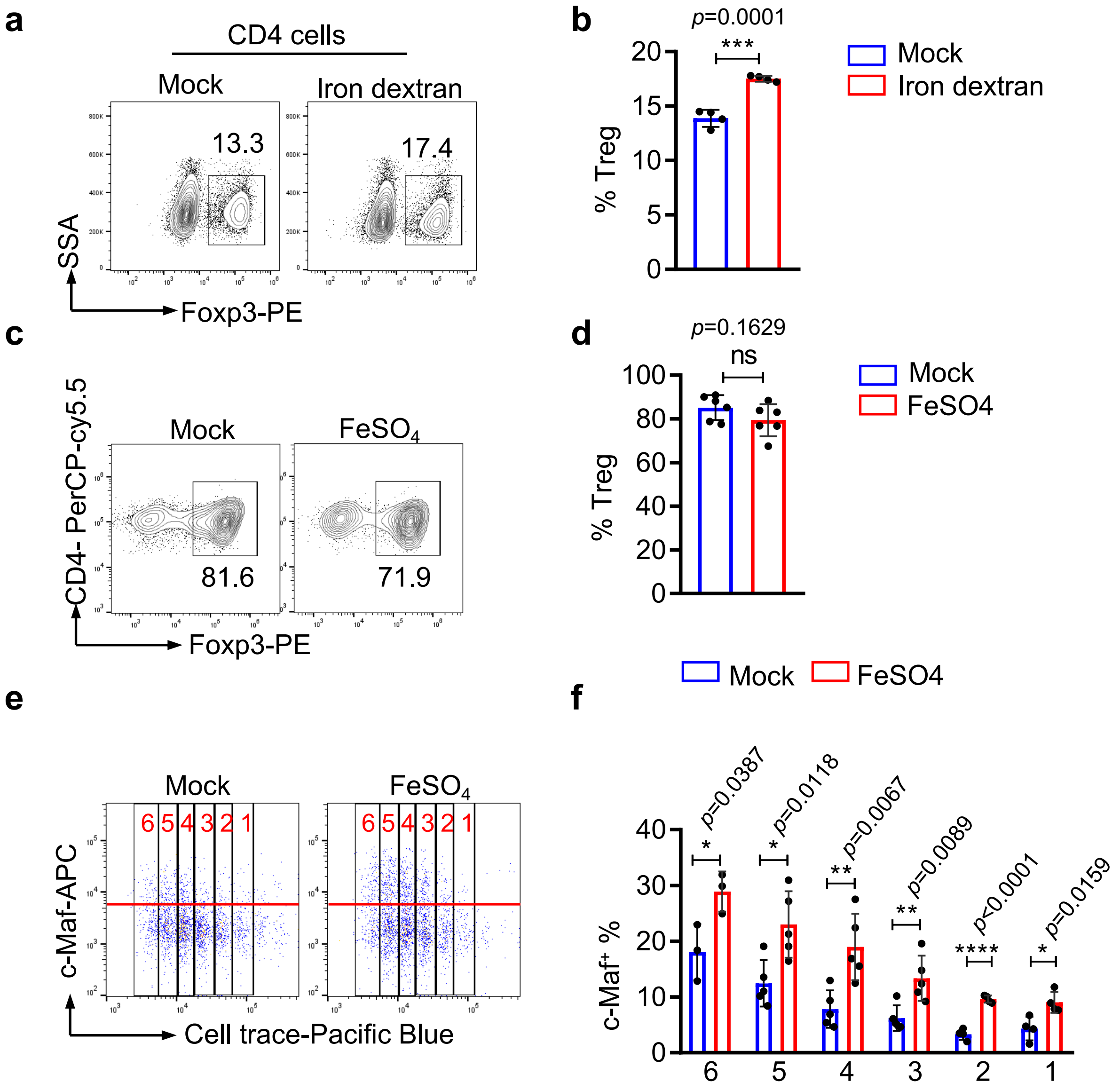

a
CD4 cells
Mock
Iron dextran
13.3
17.4
SSA
Foxp3-PE
b
p=0.0001
***
% Treg
Mock
Iron dextran
c
Mock
FeSO4
81.6
71.9
CD4- PerCP-cy5.5
Foxp3-PE
d
p=0.1629
ns
% Treg
Mock
FeSO4
e
Mock
FeSO4
6 5 4 3 2 1
c-Maf-APC
Cell trace-Pacific Blue
f
Mock
FeSO4
p=0.0387
p=0.0118
p=0.0067
p=0.0089
p<0.0001
p=0.0159
c-Maf+ %

Figure S5

Figure S5. Iron promotes the differentiation of c-Maf$^+$ Treg cells.

(**a,b**). Iron dextran treatment expanded Treg cells *in vivo*. WT C57BL/C6 mice were treated with iron dextran (37.5 mg/e.a.) by intraperitoneal injection every 5 days. Twenty-one days after the initial treatment, the ratios of Foxp3$^+$ cells were determined by intracellular staining. (**a**), Representative flow cytometric plots; (**b**), Summary of three independent experiments (n=4 mice in each condition).

(**c,d**). Iron sulfate did not promote the differentiation of Treg cells *in vitro*. Naïve CD4 T cells were isolated from the spleens of WT mice, and Treg cells were differentiated *in vitro* in the presence of TGF β and IL-2. Foxp3$^+$ cells were identified by intracellular staining. The data are representative (**c**) or a summary (**d**) of three independent experiments (n=6 samples in each condition).

(**e,f**). Iron sulfate enhanced c-Maf expression in iTreg cells independent of the enhanced cellular proliferation. Naïve CD4 T cells were isolated from the spleen and labeled with cell trace, and Treg cells were differentiated *in vitro* in the presence of 50 μM $FeSO_4$ or PBS for 4 days. c-Maf expression was determined in the cells with the indicated cell division cycles based on the cell trace intensity (**e**). The data are representative of at least three independent experiments (**f**).

(**b,d,f**) The error bars represent the standard deviation of the mean. *p<0.05, **p<0.01, ****p<0.0001 in two-tailed Student's *t test*.

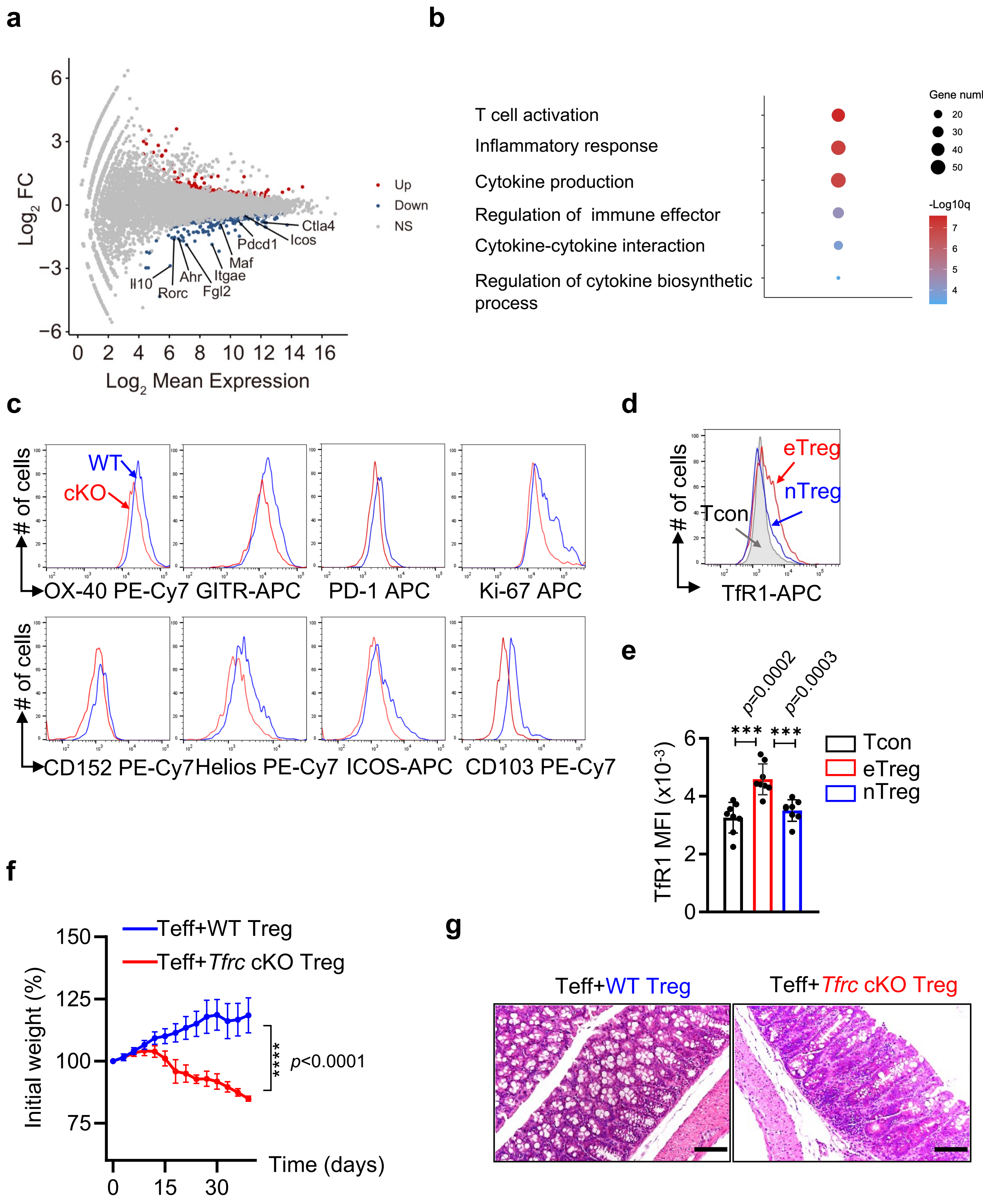
a
b
Up
Down
NS
Log2 FC
Log2 Mean Expression
Ctla4
Icos
Pdcd1
Maf
Itgae
Fgl2
Ahr
Rorc
Il10
T cell activation
Inflammatory response
Cytokine production
Regulation of immune effector
Cytokine-cytokine interaction
Regulation of cytokine biosynthetic process
Gene number
-Log10q
c
d
WT
cKO
# of cells
OX-40 PE-Cy7
GITR-APC
PD-1 APC
Ki-67 APC
CD152 PE-Cy7
Helios PE-Cy7
ICOS-APC
CD103 PE-Cy7
eTreg
nTreg
Tcon
TfR1-APC
e
p=0.0002
p=0.0003
TfR1 MFI (x10-3)
Tcon
eTreg
nTreg
f
Teff+WT Treg
Teff+Tfrc cKO Treg
Initial weight (%)
Time (days)
p<0.0001
g
Teff+WT Treg
Teff+Tfrc cKO Treg

Figure S6

Figure S6. TfR1 was essential for the effector program of Treg cells.

(**a**). TfR1 deficiency resulted in a diminished effector Treg program. YFP^+CD4^+ cells were isolated from the spleens and lymph nodes of *Tfrc*$^{fl/fl}$ *Foxp3-Cre*$^{YFP/+}$ mice (*TfR1* cKO) and *Tfrc*$^{+/+}$*Foxp3-Cre*$^{YFP/+}$ (WT) mice. RNA-seq was conducted, and differentially expressed genes were identified with DESeq-2. The data are shown as an M-A plot based on two biological replicates. Y-axis and X-axis correspond to log2FC and the baseMean value respectively. Significantly changed genes in TfR1-deficient Treg cells are highlighted in red (upregulated) or blue (downregulated) ($|LFC| \geq 0.5$, p value < 0.05, Wald test).

(**b**). Gene ontology analysis of the genes identified in (**a**) was performed using Metascape [1]. The size of the dot represents the number of enriched genes, and the color represents the p value of the indicated GO term. *p*-values are calculated based on the cumulative hypergeometric distribution, and q-values are calculated using the Benjamini-Hochberg procedure.

(**c**). The expression of effector molecules in the Treg cells identified in (**a**) was determined by flow cytometry. The data are representative of three independent experiments.

(**d,e**). TfR1 expression was increased in effector Treg cells. The expression of TfR1 in effector Treg ($CD44^{hi}CD62L^{low}$ $Foxp3^+CD4^+$), naïve Treg ($CD44^{lo}CD62L^{hi}Foxp3^+CD4^+$) and conventional CD4 ($Foxp3^-CD4^+$) cells was determined by flow cytometry. (**d**), Representative flow cytometric results; (**e**), Summary of three independent experiments (n=8 mice per group, Two-tailed *t test*, ****p*<0.001, mean ± SD.).

(**f,g**). TfR1-deficient Treg cells lost their suppressive function *in vivo*. A total of $1x10^5$ YFP^+CD25^+ cells isolated from the spleens of 6-day-old *Tfrc*$^{fl/fl}$ *Foxp3*$^{Cre\text{-}IRES\text{-}YFP/y}$ (TfR1 cKO) and *Tfrc*$^{+/+}$ *Foxp3*$^{Cre\text{-}IRES\text{-}YFP/y}$ mice (WT) were cotransferred with $4x10^5$ naïve T cells (WT $CD4^+$ $CD45RB^+CD25^-$ cells) into *Rag1*$^{-/-}$ mice. Weight loss (**f**) and inflammatory cell infiltration in the colon (**g**) were analyzed in two independent experiments, scale bars represent 100 μm. The significance of the data in **f** was determined by two-way ANOVA (****, *p*<0.0001, mean ± SEM.).

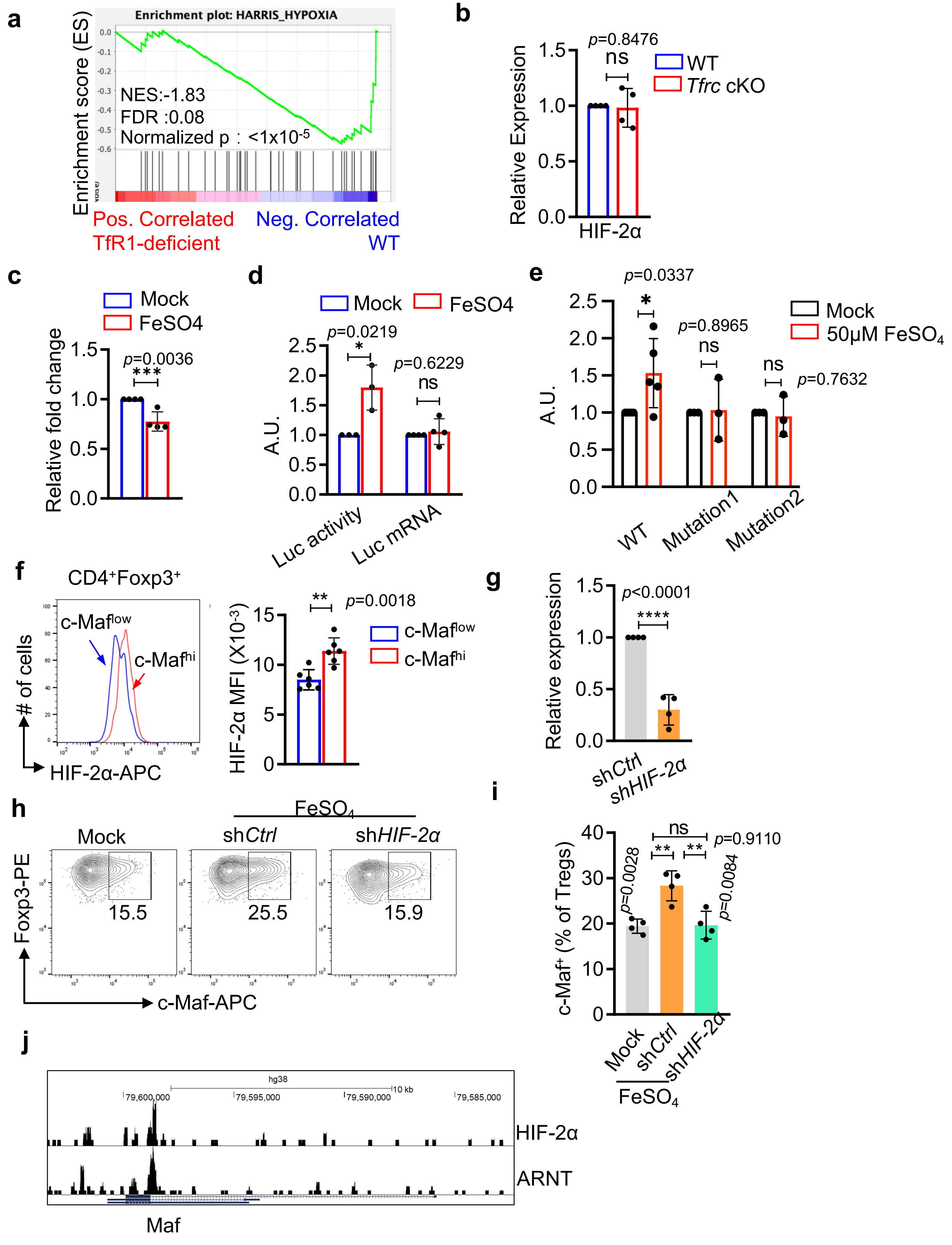

a
Enrichment plot: HARRIS_HYPOXIA
Enrichment score (ES)
NES:-1.83
FDR :0.08
Normalized p : <1x10-5
Pos. Correlated
TfR1-deficient
Neg. Correlated
WT
b
p=0.8476
ns
WT
Tfrc cKO
Relative Expression
HIF-2α
c
Mock
FeSO4
p=0.0036
***
Relative fold change
d
Mock
FeSO4
p=0.0219
*
p=0.6229
ns
A.U.
Luc activity
Luc mRNA
e
p=0.0337
*
p=0.8965
ns
ns
p=0.7632
Mock
50μM FeSO4
A.U.
WT
Mutation1
Mutation2
f
CD4+Foxp3+
c-Maflow
c-Mafhi
# of cells
HIF-2α-APC
**
p=0.0018
HIF-2α MFI (X10-3)
c-Maflow
c-Mafhi
g
p<0.0001
****
Relative expression
shCtrl
shHIF-2α
h
FeSO4
Mock
shCtrl
shHIF-2α
Foxp3-PE
15.5
25.5
15.9
c-Maf-APC
i
ns
p=0.9110
**
**
p=0.0028
p=0.0084
c-Maf+ (% of Tregs)
Mock
shCtrl
shHIF-2α
FeSO4
j
hg38
10 kb
79,600,000
79,595,000
79,590,000
79,585,000
HIF-2α
ARNT
Maf

Figure S7

Figure S7. Iron promotes c-Maf expression in Treg cells by enhancing HIF-2α translation.

(**a**). Gene expression of TfR1-deficient Treg cells (YFP^+CD4^+ cells from *Tfrc*$^{fl/fl}$ *Foxp3*$^{Cre\text{-}YFP/+}$ mice) was compared to WT Treg cells (YFP^+CD4^+ cells from *Tfrc* $^{+/+}$*Foxp3*$^{Cre\text{-}YFP/+}$ mice) by RNA-seq. GSEA was performed using the GSEA tool and MSigDB database [2]. The significance of a gene set is estimated by an empirical phenotype-based permutation test procedure.

(b). HIF-2α expression in $CD4^+YFP^+$ cells from *Tfr1*$^{fl/fl}$ *Foxp3-Cre*$^{YFP/+}$ mice (TfR1 cKO) was determined with real-time PCR and compared to that in the counterparts from *Tfrc*$^{+/+}$*Foxp3-Cre*$^{YFP/+}$ mice (n=4 mice from three independent experiments).

(c). As shown in **Figure 3h**, iTreg cells were stimulated with $FeSO_4$, and HIF-2α mRNA levels were determined. The data are a summary of four independent experiments.

(d). iTreg cells were transfected with a HIF-2α 5'-UTR luciferase reporter and were stimulated with FeSO4 for 12 hours. Luciferase activity (*left*) (n=3) and luciferase mRNA (*right*) (n=4) were determined. The data were normalized to cells without $FeSO_4$ stimulation and summarized in three independent experiments.

(**e**). As in (**d**), WT iTreg cells were transfected with either WT HIF-2α 5'-UTR reporter or mutant reporters abrogating the IRE/IRP interaction [3 4]. The data were normalized to the cells without $FeSO_4$ stimulation (n=4 and 3 for WT and mutant groups).

(f). WT Treg cells were identified as $CD4^+$ $Foxp3^+$, and HIF-2α expression in the c-Maf ^{hi} population was compared to that in the c-Maf ^{low} population. The data are representative (*left*) or a summary (*right*) of three independent experiments (n=6 mice per group).

(**g,h**). iTreg cells were transduced with indicated retrovirus and stimulated with $FeSO_4$. The expression of HIF-2α and c-Maf were determined by real-time PCR (**g**) or intracellular staining (**h-i**) five days after differentiation. (**h**), Representative results; (**i**). Summary of the percentage of c-Maf^+ cells in iTreg cells based on four independent experiments.

(**b-h**). Statistical significance was determined by two-tailed t test (*, p<0.05, **p<0.01, **** p<0.0001), and data are mean ± SD.

(**j**). HIF-2α and ARNH bind to the promoter region of c-Maf. The data were derived from the results of previously published ChIP seq experiments using the kidney epithelium cell line 786-O [5].

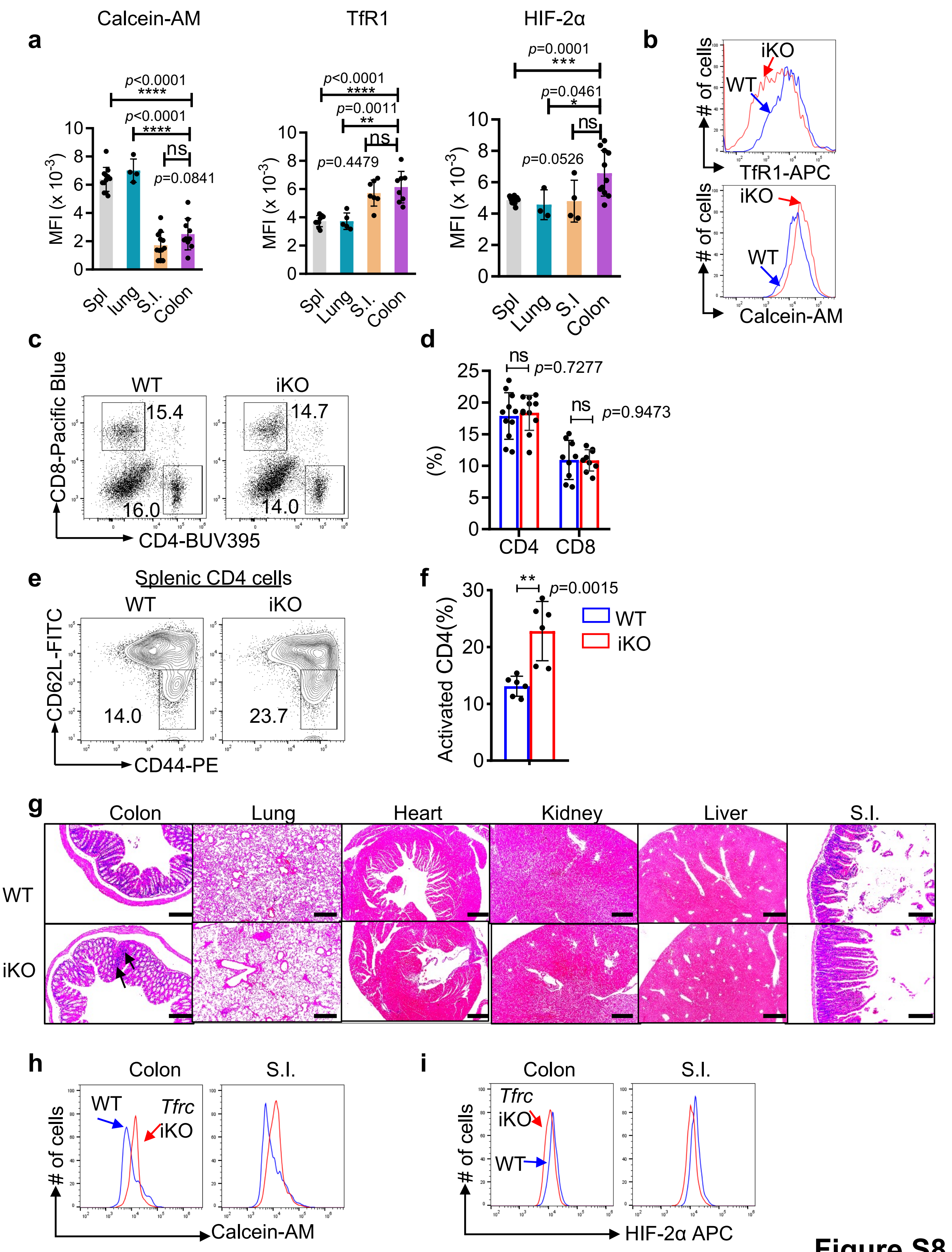
a
Calcein-AM
TfR1
HIF-2α
p<0.0001
****
p<0.0001
****
ns
p=0.0841
MFI (x 10-3)
Spl
lung
S.I.
Colon
p<0.0001
****
p=0.0011
**
ns
p=0.4479
Lung
p=0.0001
***
p=0.0461
*
ns
p=0.0526
b
iKO
WT
# of cells
TfR1-APC
Calcein-AM
c
WT
iKO
15.4
16.0
14.7
14.0
CD8-Pacific Blue
CD4-BUV395
d
ns
p=0.7277
ns
p=0.9473
(%)
CD4
CD8
e
Splenic CD4 cells
CD62L-FITC
CD44-PE
14.0
23.7
f
**
p=0.0015
Activated CD4(%)
WT
iKO
g
Colon
Lung
Heart
Kidney
Liver
S.I.
h
Colon
S.I.
Tfrc
Calcein-AM
i
HIF-2α APC

Figure S8

Figure S8. Acute depletion of TfR1 in Treg cells selectively disrupts immune tolerance in the intestine.

(**a**). $CD4^+Foxp3^+$ cells from indicated organs of WT mice were analyzed for the levels of labile iron by Calcein-AM staining (*left*) (n=12,4,12 and 10 mice for spleen, lung, small intestine and colon, respectively) or for the expression of TfR1 (*middle*) (n=9,5,8,8 mice per group for the spleen, lung, small intestine and colon, respectively) and HIF-2α (*right*) (n=15,3,4,and 11 mice per group for the spleen, lung, small intestine and colon, respectively) by flow cytometry. Note, the calcein-AM levels were inversely correlated with the levels of intracellular labile iron.

(**b**). *Foxp3*$^{eGFP\text{-}creERT2}$*Tfrc* $^{fl/fl}$ mice were treated with tamoxifen as described in Figure **4c**, and the expression of TfR1 (*top*) and the levels of labile iron (calcein-AM staining, *bottom*) in splenic Treg cells were analyzed.

(**c,d**). The ratios of CD4 and CD8 T cells in the spleens of tamoxifen-treated *Foxp3*$^{eGFP\text{-}creERT2}$*Tfrc* $^{fl/fl}$ mice were analyzed. (**c**). Representative flow cytometry analysis; (**d**). Summary of the percentages of $CD4^+$ and $CD8^+$ T cells from three independent experiments (n=11 and 9 for CD4+ cells and CD8+ cells respectively). Each dot represents one individual mouse.

(**e,f**). Splenic CD4 T cells were analyzed for the expression of CD44 and CD62L, and the effector/memory population was gated as $CD44^{hi}CD62L^{low}$. (**e**). Representative flow cytometry analysis; (**f**). Summary of the percentages of the $CD44^{hi}CD62L^{low}$ population in CD4 T cells (n=6 mice per group from three independent experiments).

(**g**). H&E staining of the indicated tissues from tamoxifen-treated *Foxp3*$^{eGFP\text{-}creERT2}$*Tfrc* $^{fl/fl}$ mice (iKO). The images are representative of three independent experiments. The arrows indicate infiltrating inflammatory cells. Scale bars represent 250 μm (colon and small intestine (S.I.) or 500 μm (lung, heart, kidney and liver).

(**h,i**). Treg cells from either the colon or small intestine (S.I.) of the tamoxifen-treated *Foxp3*$^{eGFP\text{-}creERT2}$*Tfrc* $^{fl/fl}$ mice (*Tfrc* iKO) were analyzed for the levels of labile iron (Calcein-AM staining, **h**) or HIF-2α by intracellular staining (**I**). The data are representative of three independent experiments.

(**a,d,f**) Statistical significance was determined by two-tailed students' *t* test, and the data are mean ± SD. *p<0.05, **p<0.01, ***p<0.001,****p<0.0001.

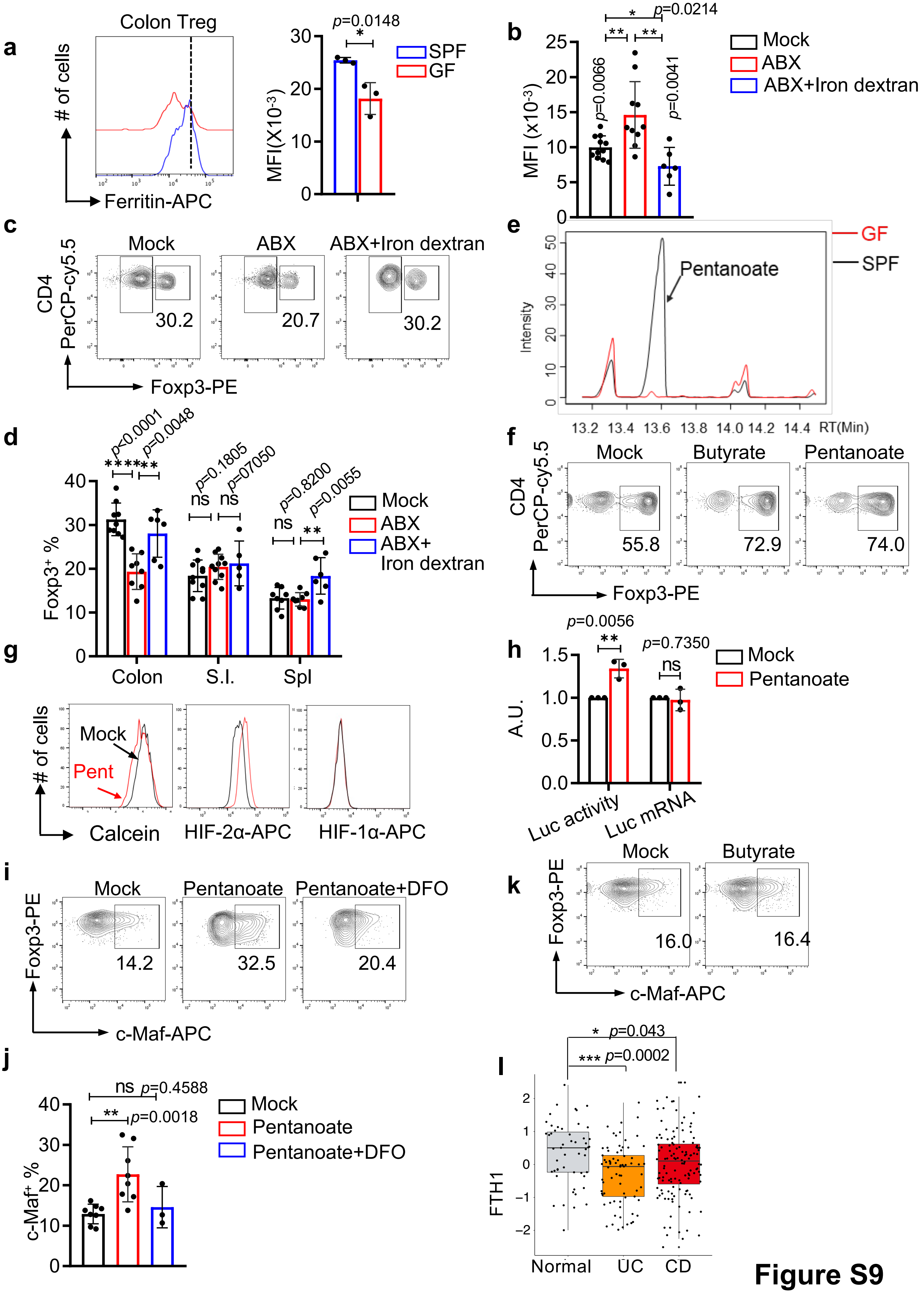
a
Colon Treg
# of cells
Ferritin-APC
p=0.0148
*
SPF
GF
MFI(X10-3)
b
p=0.0214
MFI (x10-3)
p=0.0066
p=0.0041
Mock
ABX
ABX+Iron dextran
c
Mock
ABX
ABX+Iron dextran
CD4 PerCP-cy5.5
30.2
20.7
30.2
Foxp3-PE
d
p<0.0001
p=0.0048
p=0.1805
p=07050
p=0.8200
p=0.0055
ns
Foxp3+ %
Colon
S.I.
Spl
Mock
ABX
ABX+ Iron dextran
e
GF
SPF
Pentanoate
Intensity
RT(Min)
f
Mock
Butyrate
Pentanoate
CD4 PerCP-cy5.5
55.8
72.9
74.0
Foxp3-PE
g
# of cells
Mock
Pent
Calcein
HIF-2α-APC
HIF-1α-APC
h
p=0.0056
p=0.7350
ns
A.U.
Luc activity
Luc mRNA
Mock
Pentanoate
i
Mock
Pentanoate
Pentanoate+DFO
Foxp3-PE
14.2
32.5
20.4
c-Maf-APC
k
Mock
Butyrate
Foxp3-PE
16.0
16.4
c-Maf-APC
j
ns p=0.4588
** p=0.0018
c-Maf+ %
Mock
Pentanoate
Pentanoate+DFO
l
* p=0.043
*** p=0.0002
FTH1
Normal
UC
CD

Figure S9

Figure S9. Pentanoate enhances iron uptake and promotes the expression of HIF-2α and c-Maf.

(**a**). Ferritin expression was determined in colonic Treg cells from either SPF or germ-free mice (n=3 mice).

(**b**). Labile iron in colonic Treg cells was determined by Calcein-AM staining (n=11, 10, and 6 mice for control, ABX, and ABX+Iron dextran group respectively).

(**c,d)**. Colonic Tregs were determined by Foxp3 staining. (**c**), Representative flow cytometry plots(pregated on $CD45^{+}CD4^{+}$ cells). (**d**), Summary of the ratios of colonic Tregs from three independent experiments, and each dot represents one individual mouse.

(**e**). As in Figure **5d**, metabolites from the caeca of SPF and germ-free (GF) mice were analyzed by GC–MS. The data are representative of four independent experiments.

(f). Two days after iTreg differentiation, the expression of Foxp3 was determined. The data are representative of three independent experiments.

(**g**). As in (**f**), the expression of HIF-2α and HIF-1α in iTreg cells was determined by flow cytometry staining. Labile iron levels were determined by calcein-AM staining.

(**h**). WT iTreg cells were transfected with HIF-2α-5UTR reporter and subsequently treated with 1 mM sodium pentanoate for 12 hours. The data are a summary of three independent experiments.

(**i,j**). As in (**d**), iTreg cells were treated with pentanoate (1 mM) and with or without DFO (10 μM) for 3 days, and the expression of c-Maf was determined. The data are representative (**i**) or (**j**) summary of three independent experiments (n=8,8, and 3 mice for control, pentanoate, and pentanoate + DFO group respectively).

(**k**). As in (**d**), c-Maf expression in iTreg cells was determined 3 days after sodium butyrate treatment.

(**l**). As in **Figure 7i**, *FTH1* expression in the intestine was determined with RNA-seq in healthy individuals (normal) and patients with active CD or UC. Data are profiled from the previously published HPM2 cohort (CD, n= 43; UC, n= 25; normal, n= 22). The box plots show the median and interquartile range (IQR), with the upper and lower whiskers representing 1.5 times the IQR above the upper quartile and below the lower quartile, respectively. *, $p<0.05$, ***, $p<0.001$ in linear regression analysis.

(a,b,d,h,j). Statistical significance was determined by two-tailed Student's *t* test (**$p<0.01$, ****$p<0.0001$), and data shown are mean ± SD.

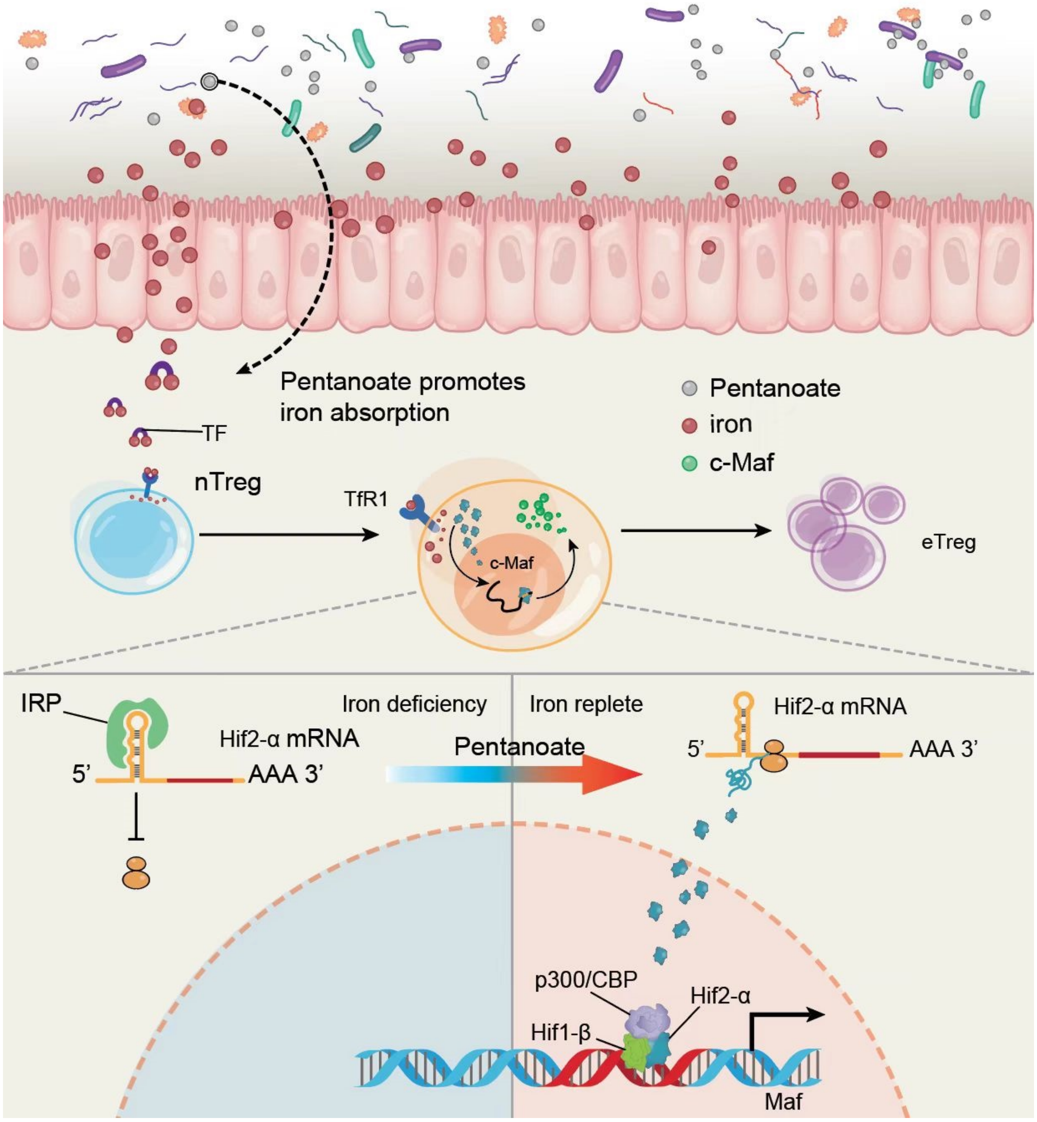

Pentanoate promotes
iron absorption
Pentanoate
iron
c-Maf
TF
nTreg
TfR1
c-Maf
eTreg
IRP
Iron deficiency
Iron replete
Hif2-α mRNA
5'
AAA 3'
Pentanoate
Hif2-α mRNA
5'
AAA 3'
p300/CBP
Hif2-α
Hif1-β
Maf

Figure S10

Figure S10. A proposed model of this study.

Commensal microbiota produces pentanoate, which enhances iron uptake in the intestine and boosts iron procurement by colonic Treg cells. Elevated intracellular iron levels prevent IRPs from binding to the 5'UTR of HIF-2α mRNA and enhance the translation of this transcription factor in Treg cells. HIF-2α then induces c-Maf expression together with HIF1β and maintains immune tolerance in the intestine. Iron deficiencies caused by the reduced expression of TfR1, absence of commensal microbiota, or dysbiosis result in the reduction in regulatory T cells in the intestine and autoinflammation in the intestine.

a. Gating strategy in the colon

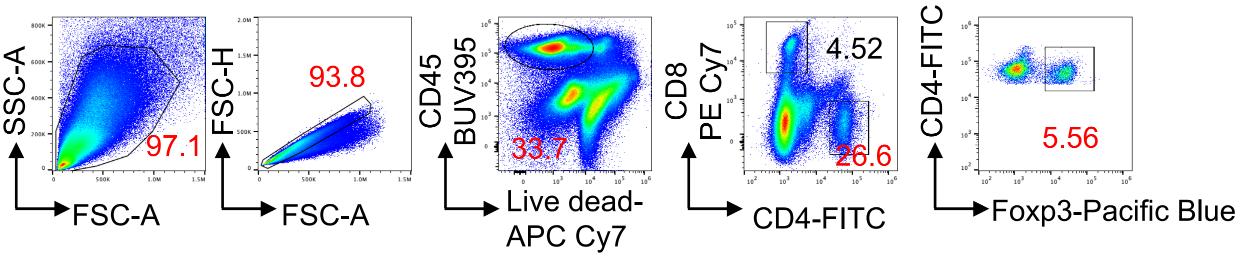

b. Gating strategy for Treg cells in the colon of *Tfrc*$^{+/+}$*Foxp3*$^{YFP\text{-}Cre/+}$ mice

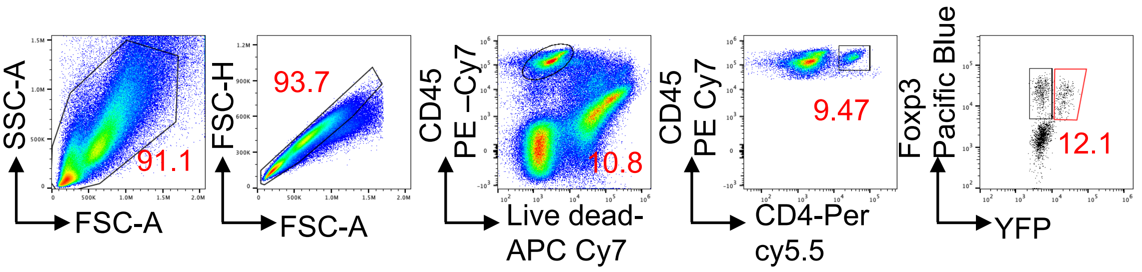

c. Gating strategy for Treg cells in bone marrow transplantation model.

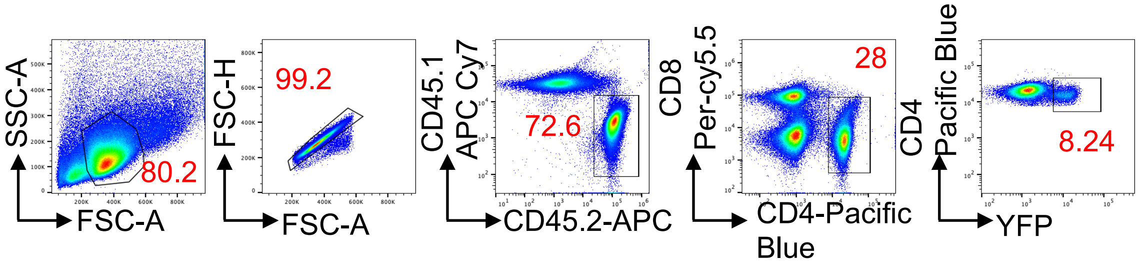

d. Gating strategy for induced Treg cells *in vitro*.

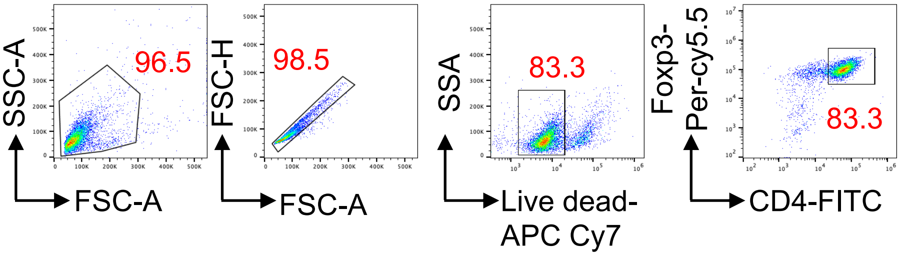

Figure S11

Figure S11. Gating strategies for flow cytometric analysis

a-d. Lymphocytes were first identified by FSC-A/SSC-A gating, followed by singlet isolation based on FCS-H/FCS-A doublet-exclusion gating.

a. Gating strategy in the colon. Cells retrieved from the mouse colon were pre-gated on singlet cells by sequentially applying a Live/Dead Fixable dye vs CD45 gate. CD4 T cells were identified as $CD45^{+}CD4^{+}CD8^{-}$. The percentage of Treg cells was assessed for the expression of Foxp3 in total CD4 T cells.

b. Gating strategy for Treg cells in the colon of $Tfrc^{+/+}Foxp3^{YFP\text{-}Cre/+}$ mice. As in **a**, total CD4 T cells in $Tfrc^{+/+}Foxp3^{YFP\text{-}Cre/+}$ mice were pre-gated on live $CD45^{+}$ lymphocytes. Then Treg cells were divided into two populations based on the expression of Foxp3 and YFP.

c. Gating strategy for Treg cells in the bone marrow transplantation model. *Rag1-/-* mice were reconstituted with *Tfrc* cKO ($Tfrc^{fl/fl}$ $Foxp3^{YFP\text{-}IRES\text{-}Cre/y}$ mice, $CD45.2^{+}$) or WT ($Tfrc^{+/+}Foxp3^{YFP\text{-}IRES\text{-}Cre/y}$ mice $CD45.2^{+}$) bone marrow cells mixed with B6.SJL ($CD45.1^{+}$) bone marrow. Eight weeks later, transferred $CD45.2^{+}$ lymphocytes in the recipients were identified by singlet cell gating and CD45.2+ gating. Tester CD4 T cells were defined as $CD45.2^{+}CD4^{+}CD8^{-}$. After gating $CD4^{+}$ T cells, Treg cells were assessed based on YFP expression.

d. Gating strategy for induced Treg cells *in vitro*. For Treg cells cultured *in vitro*, lymphocytes were pre-gated on live singlet cells by FCS-H vs FCS-A gating and Live/Dead Fixable dye vs SSA gating. After gating on singlet cells, Treg cells were defined as $CD4^{+}$ $Foxp3^{+}$.

References

1. Zhou, Y.Y. *et al.* Metascape provides a biologist-oriented resource for the analysis of systems-level datasets. *Nature Communications* **10** (2019).

2. Subramanian, A. *et al.* Gene set enrichment analysis: a knowledge-based approach for interpreting genome-wide expression profiles. *Proc Natl Acad Sci U S A* **102**, 15545-15550 (2005).

3. Sanchez, M., Galy, B., Muckenthaler, M.U. & Hentze, M.W. Iron-regulatory proteins limit hypoxia-inducible factor-2alpha expression in iron deficiency. *Nat Struct Mol Biol* **14**, 420-426 (2007).

4. Zimmer, M. *et al.* Small-molecule inhibitors of HIF-2a translation link its 5'UTR iron-responsive element to oxygen sensing. *Mol Cell* **32**, 838-848 (2008).

5. Schodel, J. *et al.* Common genetic variants at the 11q13.3 renal cancer susceptibility locus influence binding of HIF to an enhancer of cyclin D1 expression. *Nature Genetics* **44**, 420-U229 (2012).
