# Supplementary material for: Microbiota-assisted iron uptake promotes immune tolerance in the intestine
Source: Nat Commun. 2023 May 15;14:2790. doi: 10.1038/s41467-023-38444-2 (PMC10185671; doi:10.1038/s41467-023-38444-2)
Supplement: Supplementary file 3 — Description of Additional Supplementary Files [file 41467_2023_38444_MOESM3_ESM.pdf]

Description of Additional Supplementary Files

Supplementary Data 1. PRISM dataset used in this study.

Supplementary Data 2. HMP2 dataset used in this study.

Supplementary Data 3. Primers used in this study.
