# Supplementary material for: Microbiota-assisted iron uptake promotes immune tolerance in the intestine
Source: Nat Commun. 2023 May 15;14:2790. doi: 10.1038/s41467-023-38444-2 (PMC10185671; doi:10.1038/s41467-023-38444-2)
Supplement: Supplementary file 7 — Reporting Summary [file 41467_2023_38444_MOESM7_ESM.pdf]

Corresponding author(s): Xing Chang

Last updated by author(s): Apr 20, 2023

Reporting Summary

Nature Portfolio wishes to improve the reproducibility of the work that we publish. This form provides structure for consistency and transparency in reporting. For further information on Nature Portfolio policies, see our Editorial Policies and the Editorial Policy Checklist.

Statistics

For all statistical analyses, confirm that the following items are present in the figure legend, table legend, main text, or Methods section.

n/a	Confirmed	
☐	☒	The exact sample size (*n*) for each experimental group/condition, given as a discrete number and unit of measurement
☐	☒	A statement on whether measurements were taken from distinct samples or whether the same sample was measured repeatedly
☐	☒	The statistical test(s) used AND whether they are one- or two-sided<br>*Only common tests should be described solely by name; describe more complex techniques in the Methods section.*
☐	☒	A description of all covariates tested
☐	☒	A description of any assumptions or corrections, such as tests of normality and adjustment for multiple comparisons
☐	☒	A full description of the statistical parameters including central tendency (e.g. means) or other basic estimates (e.g. regression coefficient) AND variation (e.g. standard deviation) or associated estimates of uncertainty (e.g. confidence intervals)
☐	☒	For null hypothesis testing, the test statistic (e.g. *F*, *t*, *r*) with confidence intervals, effect sizes, degrees of freedom and *P* value noted<br>*Give P values as exact values whenever suitable.*
☒	☐	For Bayesian analysis, information on the choice of priors and Markov chain Monte Carlo settings
☒	☐	For hierarchical and complex designs, identification of the appropriate level for tests and full reporting of outcomes
☐	☒	Estimates of effect sizes (e.g. Cohen's *d*, Pearson's *r*), indicating how they were calculated

Our web collection on statistics for biologists contains articles on many of the points above.

Software and code

Policy information about availability of computer code

Data collection	Beckman CytoFLEX Analyzer
Data analysis	GraphPad Prism (v9), FlowJo(v10), NIST MS Search 2.3

For manuscripts utilizing custom algorithms or software that are central to the research but not yet described in published literature, software must be made available to editors and reviewers. We strongly encourage code deposition in a community repository (e.g. GitHub). See the Nature Portfolio guidelines for submitting code & software for further information.

Data

Policy information about availability of data

All manuscripts must include a data availability statement. This statement should provide the following information, where applicable:

- Accession codes, unique identifiers, or web links for publicly available datasets
- A description of any restrictions on data availability
- For clinical datasets or third party data, please ensure that the statement adheres to our policy

Source data are provided with this paper. scRNA-seq and bulk RNA sequencing datasets have been deposited in the GEO under accession number GEO: GSE195607. The mass spectra data of PRISM was deposited at metabolomics workbench study ST000923. The Taxonomic and functional profiles for metabolomes (MBX) and host transcriptomes (HTX) were deposited at HMP2 which could been downloaded at http://ibdmdb.org in July 2020. The authors declare that all data supporting the findings of this study are available within the paper and its Supplementary Information files or from the corresponding author upon reasonable request.

Human research participants

Policy information about studies involving human research participants and Sex and Gender in Research.

Reporting on sex and gender	Clinical data used for IBD analysis were obtained from HPM2 and PRISM, sex and gender of specimens were presented in Supplementary dataset 1 and 2.
Population characteristics	Population characteristics were indicated in Supplementary dataset 1 and 2.
Recruitment	Clinical data included in this paper were downloaded from HPM2 or PRSIM database.The authors do not see any potential bias in the generation or interpretation of the data reported in this study.
Ethics oversight	HMP2 was reviewed by the Institutional Review Boards at each sampling site:overall Partners Data Coordination (IRB #2013P00215); MGH Adult cohort (IRB#2004P001067);MGH Paediatrics (IRB# 2014P001115);Emory (IRB#00071468);Cincinnati Children's Hospital Medical Center (2013-7586);and Cedars-Sinai Medical Center (3358/ CR00011696).PRISM was reviewed and approved by the Partners Human Research Committee(ref.2004-001067).

Note that full information on the approval of the study protocol must also be provided in the manuscript.

Field-specific reporting

Please select the one below that is the best fit for your research. If you are not sure, read the appropriate sections before making your selection.

[x] Life sciences   [ ] Behavioural & social sciences   [ ] Ecological, evolutionary & environmental sciences

For a reference copy of the document with all sections, see nature.com/documents/nr-reporting-summary-flat.pdf

Life sciences study design

All studies must disclose on these points even when the disclosure is negative.

Sample size	No statistical method was used to predetermine the sample size for experiments. Sample size was based on experimental feasibility, sample availability and according to published literature. See specific legends for details.
Data exclusions	No data was excluded from analyses.
Replication	Replicates were used in all experiments as noted in figure legends, and all experiments were successfully repeated at least three times.
Randomization	Mice were age and sex-matched and randomized where appropriate (e.g. prior to initiating treatment for matched conditions). Mice used in antibiotics or DSS treatment were administrated to antibiotics or DSS first, then randomly assigned to control or pentanoate groups. For other experiments, mice were randomized and evenly distributed across both male and female littermates in cages. In experiments involving transgenic mice, littermates with different genotypes were cohoused for the duration of experiments. Allocation into experimental groups was random.
Blinding	Histopathological analysis was single-blinded. Investigators were no blinded to treatment groups or data analysis, as knowledge of this information was essential to conduct the studies.

Reporting for specific materials, systems and methods

We require information from authors about some types of materials, experimental systems and methods used in many studies. Here, indicate whether each material, system or method listed is relevant to your study. If you are not sure if a list item applies to your research, read the appropriate section before selecting a response.

Materials & experimental systems

n/a	Involved in the study	
[ ]	[x]	Antibodies
[ ]	[x]	Eukaryotic cell lines
[x]	[ ]	Palaeontology and archaeology
[ ]	[x]	Animals and other organisms
[x]	[ ]	Clinical data
[x]	[ ]	Dual use research of concern

Methods

n/a	Involved in the study	
[x]	[ ]	ChIP-seq
[ ]	[x]	Flow cytometry
[x]	[ ]	MRI-based neuroimaging

Antibodies

Antibodies used

TfR1-APC (R17217, Invitrogen, 17071182), CD44-APC (IM7, Biolegend, 103012), CD44-FITC (IM7, Biolegend,103005), CD62L-PE/Cy7 (MEL-14, Invitrogen, 25062182), Foxp3-PE (FJK-16s, Invitrogen, 12577382), CD4-PerCP/Cy5.5 (GK1.5, Biolegend, 100434), Foxp3-Pacific Blue (FJK-16s, Invitrogen, 48577382), CD25-APC (PC61, Biolegend, 102012), c-Maf-APC (sym0F1, Invitrogen, 50985582), HIF-2α-APC (ep190b, Invitrogen, MA5-16021), HIF-1α-APC (Mgc3, Invitrogen, 17-7528-82), anti-CD16/32 (2.4G2, BD Pharmingen™, 553142), IL4-PE/Cy7 (11B11, Biolegend, 504117), IL-17A-APC (TC11-18H10.1, Biolegend, 506911), INF-γ-APC (XMG1.2, Biolegend, 505810), CD4-FITC(GK1.5, BD Pharmingen™, 557307), CD11b-PerCP/Cy5.5 (M1/70, Biolegend,101228), CD19-PE/Cy7 (6D5, Biolegend,115520), CD8-APC (53-6.7, Biolegend, 100724), CD4-Pacific Blue (GK1.5, Biolegend,100428), CD44-PE (IM7, Biolegend, 103024), OX-40-PE/Cy7 (OX-86, Biolegend, 119415), GITR-APC (DTA-1, Biolegend, 126312), PD-1-APC (J43, eBioscience™, 17998582), Ki-67-APC (16A8, Biolegend, 652406), CD152-PE/Cy7 (UC10-4B9, Biolegend, 106314), Helios-PE/Cy7 (22F6, Biolegend, 137236), ICOS-APC (7E.17G9, Invitrogen, 17994282), CD103- PE/Cy7 (2E7, Biolegend, 121425), Ahr-PE (4MEJJ, Invitrogen, 12-5925-82), T-bet-PE (4B10, eBioscience,12-5825-80), ROR gamma (t) -PE (B2D, eBioscience™,12-6981-82),Gata3 PE-Cy7 (TWAJ, eBioscience™, 25-9966-42), ferritin (EPR3004Y, abcam, ab75973).

Validation

Antibodies used in this study are commercially available and are validated by the manufacturers, related information are available from their website:

Rat anti mouse TfR1-APC (R17217, Invitrogen, 17071182) /flow-cytometry
https://www.thermofisher.cn/cn/zh/antibody/product/CD71-Transferrin-Receptor-Antibody-clone-R17217-RI7-217-1-4-Monoclonal/17-0711-82
Collins N,et al.Cell.2019 Aug 22;178(5):1088-1101.e15. doi: 10.1016/j.cell.2019.07.049.

Rat anti mouse /human CD44-APC (IM7, Biolegend, 103012)/flow-cytometry
https://www.biolegend.com/en-us/products/apc-anti-mouse-human-cd44-antibody-312
Dai B, et al. 2012. Mol Ther. 20:1800.

Rat anti mouse /human CD44-FITC (IM7, Biolegend,103005)/flow-cytometry
https://www.biolegend.com/en-us/products/fitc-anti-mouse-human-cd44-antibody-314
Kamijo S, et al. 2013. J Immunol. 190:4489.

Rat anti mouse CD62L-PE/Cy7 (MEL-14, Invitrogen, 25062182)/flow-cytometry
https://www.thermofisher.cn/cn/zh/antibody/product/CD62L-L-Selectin-Antibody-clone-MEL-14-Monoclonal/25-0621-82
Dean Franckaert,et al. Immunol Cell Biol. 2015 Apr;93(4):417-23. doi: 10.1038/icb.2014.108. Epub 2014 Dec 23.

Rat anti Bovine/Dog/Cat/Mouse/Pig/Rat Foxp3-PE (FJK-16s, Invitrogen, 12577382)/flow-cytometry
https://www.thermofisher.cn/cn/zh/antibody/product/FOXP3-Antibody-clone-FJK-16s-Monoclonal/12-5773-82
Valeria Roca, et al. Reproduction.2009 Oct;138(4):733-42. doi: 10.1530/REP-09-0171. Epub 2009 Jul 24.

Rat anti mouse CD4-PerCP/Cy5.5 (GK1.5, Biolegend, 100434)/flow-cytometry
https://www.biolegend.com/en-us/products/percp-cyanine5-5-anti-mouse-cd4-antibody-4220
Chen J, et al. 2014. Cell Res. 24:1050.

Rat anti Bovine/Dog/Cat/Mouse/Pig/Rat Foxp3-Pacific Blue (FJK-16s, Invitrogen, 48577382)/flow-cytometry
https://www.thermofisher.cn/cn/zh/antibody/product/FOXP3-Antibody-clone-FJK-16s-Monoclonal/48-5773-82
Michael Pritsch,et al. J Immunol Res. 2016;2016:3576028. doi: 10.1155/2016/3576028. Epub 2016 Apr 27.

Rat anti mouse CD25-APC (PC61, Biolegend, 102012)/flow-cytometry
https://www.biolegend.com/en-us/products/apc-anti-mouse-cd25-antibody-420
Oomizu S, et al. 2012. Clin Immunol. 143:51.

Mouse anti mouse/human c-Maf-APC (sym0F1, Invitrogen, 50985582)/flow-cytometry
https://www.thermofisher.cn/cn/zh/antibody/product/c-MAF-Antibody-clone-sym0F1-Monoclonal/50-9855-82
Dominik Aschenbrenner, et al. Nat Immunol. 2018 Oct;19(10):1126-1136. doi: 10.1038/s41590-018-0200-5. Epub 2018 Sep 10.

Mouse anti Bovine/Hamster/Human/Mouse/Rat HIF-2α-APC (ep190b, Invitrogen, MA5-16021)/flow-cytometry
https://www.thermofisher.cn/cn/zh/antibody/product/HIF-2-alpha-Antibody-clone-ep190b-Monoclonal/MA5-16021

Mouse anti mouse/human HIF-1α-APC (Mgc3, Invitrogen, 17-7528-82)/flow-cytometry
https://www.thermofisher.cn/cn/zh/antibody/product/HIF-1-alpha-Antibody-clone-Mgc3-Monoclonal/17-7528-82

anti mouse anti-CD16/32 (2.4G2, BD Pharmingen™, 553142)/flow-cytometry
https://www.bdbiosciences.com/en-us/products/reagents/flow-cytometry-reagents/research-reagents/single-color-antibodies-ruo/purified-rat-anti-mouse-cd16-cd32-mouse-bd-fc-block.553142
Araujo-Jorge T, et al. An Fc gamma RII-, Fc gamma RIII-specific monoclonal antibody (2.4G2) decreases acute Trypanosoma cruzi infection in mice. Infect Immun. 1993; 61(11):4925-4928.

Rat anti mouse IL4-PE/Cy7 (11B11, Biolegend, 504117)/flow-cytometry

https://www.biolegend.com/en-us/products/pe-cyanine7-anti-mouse-il-4-antibody-7119
Wang W, et al. 2007. J. Immunol. 178:4885.

Rat anti mouse IL-17A-APC (TC11-18H10.1, Biolegend, 506911)/flow-cytometry
https://www.biolegend.com/en-us/products/alexa-fluor-647-anti-mouse-il-17a-antibody-3536
Kawashima H, et al. 2013. J Immunol. 191:3614.

Rat anti mouse NF-γ-APC (XMG1.2, Biolegend, 505810)/flow-cytometry
https://www.biolegend.com/en-us/products/apc-anti-mouse-ifn-gamma-antibody-993
Lee J, et al. 2007. Nat Immunol. 8:181.

Rat anti mouse CD4-FITC(GK1.5, BD Pharmingen™, 557307)/flow-cytometry
https://www.bdbiosciences.com/en-us/products/reagents/flow-cytometry-reagents/research-reagents/single-color-antibodies-ruo/fitc-rat-anti-mouse-cd4.557307
Frederickson GG, Basch RS. L3T4 antigen expression by hemopoietic precursor cells. J Exp Med. 1989; 169(4):1473-1478.

Rat anti mouse/Human/Cynomolgus/Rhesus CD11b-PerCP/Cy5.5 (M1/70, Biolegend,101228)/flow-cytometry
https://www.biolegend.com/en-us/products/percp-cyanine5-5-anti-mouse-human-cd11b-antibody-4257
Sharif O, et al. 2013. J Immunol. 190:5640.

Rat anti mouse CD19-PE/Cy7 (6D5, Biolegend,115520)/flow-cytometry
https://www.biolegend.com/en-us/products/pe-cyanine7-anti-mouse-cd19-antibody-1907
Morgado P, et al. 2014. Infect Immun . 82:4047.

Rat anti mouse CD8-APC (53-6.7, Biolegend, 100724)/flow-cytometry
https://www.biolegend.com/en-us/products/alexa-fluor-647-anti-mouse-cd8a-antibody-2699
Kao C, et al. 2005. Int. Immunol. 17:1607.

Rat anti mouse CD4-Pacific Blue (GK1.5, Biolegend,100428)/flow-cytometry
https://www.biolegend.com/en-us/products/pacific-blue-anti-mouse-cd4-antibody-3316
Dialynas DP, et al. 1983. J. Immunol. 131:2445.
Rat anti mouse/human CD44-PE (IM7, Biolegend, 103024)/flow-cytometry
https://www.biolegend.com/en-us/products/pe-anti-mouse-human-cd44-antibody-2206
Wang XY, et al. 2008. Blood 111:2436.

Rat anti mouse OX-40-PE/Cy7 (OX-86, Biolegend, 119415)/flow-cytometry
https://www.biolegend.com/en-us/products/pe-cyanine7-anti-mouse-cd134-ox-40-antibody-12101
Al-Shamkhani A, et al. 1996. Eur. J. Immunol. 26:1695.

Rat anti mouse GITR-APC (DTA-1, Biolegend, 126312)/flow-cytometry
https://www.biolegend.com/en-us/products/apc-anti-mouse-cd357-gitr-antibody-4646
Alissafi T, et al. 2020. Cell Metabolism. 32(4):591-604.e7.

Armenian hamster anti mouse PD-1-APC (J43, eBioscience™, 17998582)/flow-cytometry
https://www.thermofisher.cn/cn/zh/antibody/product/CD279-PD-1-Antibody-clone-J43-Monoclonal/17-9985-82
Mohammed Javeed I Ansari, et al.J Exp Med. 2003 Jul 7;198(1):63-9. doi: 10.1084/jem.20022125.

652406 Ki-67-APC (16A8, Biolegend, 652406)/flow-cytometry
https://www.biolegend.com/en-us/products/apc-anti-mouse-ki-67-antibody-8447
Serr I, et al. 2016. Nat Commun. 7:10991.

Armenian Hamster anti mouse CD152-PE/Cy7(UC10-4B9, Biolegend, 106314)/flow-cytometry
https://www.biolegend.com/en-us/products/pe-cyanine7-anti-mouse-cd152-antibody-10318
Waterhouse P, et al. 1995. Science 270:985.

Armenian Hamster anti mouse Helios-PE/Cy7 (22F6, Biolegend, 137236)/flow-cytometry
https://www.biolegend.com/en-us/products/pe-cyanine7-anti-mouse-human-helios-antibody-12089
Thornton AM, et al. 2010. J. Immunol. 184:1.

Rat anti mouse ICOS-APC (7E.17G9, Invitrogen, 17994282)/flow-cytometry
https://www.thermofisher.cn/cn/zh/antibody/product/CD278-ICOS-Antibody-clone-7E-17G9-Monoclonal/17-9942-82
Katharina Essig, et al.Nat Commun. 2018 Sep 19;9(1):3810. doi: 10.1038/s41467-018-06184-3.

Armenian Hamster anti mouse CD103- PE/Cy7 (2E7, Biolegend, 121425)/flow-cytometry
https://www.biolegend.com/en-us/products/pe-cyanine7-anti-mouse-cd103-antibody-9899
LeFrancois L, et. al, 1994. Eur. J. Immunol. 24:635.

Rat anti mouse Ahr-PE (4MEJJ, Invitrogen, 12-5925-82)/flow-cytometry
https://www.thermofisher.cn/antibody/primary/query/12-5925-82

Luis Felipe Campesato, et al. Nat Commun. 2020 Aug 11;11(1):4011. doi: 10.1038/s41467-020-17750-z.

Rabbit anti Mouse/Rat/Human ferritin (EPR3004Y, abcam, ab75973) was used for Flow Cyt (Intra) with a diution 1:100, the antibody can recongnize human Ferritin aa 50-150.

Eukaryotic cell lines

Policy information about cell lines and Sex and Gender in Research

Cell line source(s)	Plat-E cell line was purchased from ATCC.
Authentication	Plat-E cell line was not authenticated.
Mycoplasma contamination	The particular cells in the manuscript were not tested for mycoplasma, but our laboratory periodically test tissue culture facility for mycoplasma contamination in general.
Commonly misidentified lines (See ICLAC register)	No commonly misidentified lines were used in this study.

Animals and other research organisms

Policy information about studies involving animals; ARRIVE guidelines recommended for reporting animal research, and Sex and Gender in Research

Laboratory animals	Foxp3YFP-IRES-Cre, Foxp3eGFP-creERT2, Rag1-/-, and C57BL/6 SJL (CD45.1) mice were obtained from Jackson laboratory. Tfrcfl/fl mice were obtained from National Resource Center of Model Mice (Nanjing, China) and backcrossed to C57BL/6 background for over ten generations. Tfrc cKO mice was generated by crossing Tfrcfl/fl mice with Foxp3YFP-IRES-Cre mice. All mice were maintained under specific pathogen free conditions with a 12-h light, 12-h dark cycle, 30%-70% humidity, temperatures of 21.1-22.2°C, and given free access to food and water.<br>Germ-free mice were obtained and housed in germ-free isolators from National Resource Center of Model Mice (Nanjing, China).<br>Except for Tfrcfl/fl Foxp3YFP-IRES-Cre/y mice (referred to as Tfrc cKO mice), age-matched mice used for all experiments in this study were from 6 to 8 weeks of age. Tfrc cKO mice, that exhibited signs of systemic inflammation, were analyzed within 4 weeks of age.
Wild animals	No wild animals were used in this study.
Reporting on sex	Both female and male mice were included in this study. For chronic IBD model, 6-week-old female mice were administrated with DSS.
Field-collected samples	No field-collected samples were used in this study.
Ethics oversight	All experiments performed in this study were approved by the institutional biomedical research ethics committee of the Westlake University.

Note that full information on the approval of the study protocol must also be provided in the manuscript.

Flow Cytometry

Plots

Confirm that:

- [x] The axis labels state the marker and fluorochrome used (e.g. CD4-FITC).
- [x] The axis scales are clearly visible. Include numbers along axes only for bottom left plot of group (a 'group' is an analysis of identical markers).
- [x] All plots are contour plots with outliers or pseudocolor plots.
- [x] A numerical value for number of cells or percentage (with statistics) is provided.

Methodology

Sample preparation	Intestines were collected and incubated in pre-digestion buffer (1 mM DTT, 10 mM EDTA and 10 mM HEPES in PBS) at 37 °C for 30 min to remove epithelial cells. Remaining tissues were dissociated in digestion buffer containing 1 mg/mL collagenase D (Worthington), 20 μg/mL DNase I (Roche), and 10% FBS (Hyclone) with constant stirring at 37 °C for 30 min. Mononuclear cells were then collected at the interface of a 40%–70% Percoll gradient (GE Healthcare).<br>Other organoids, including liver and lung, were minced with dissection scissors, and incubated with RPMI 1640 medium supplemented with 1 mg/mL collagenase D, 20 μg/mL DNase I, and 10% FBS on a shaker at 37 °C for 30 min,followed by Percoll gradient centrifugation.
Instrument	CytoFLEX LX (Beckman) was used for flow cytometry anlysis.

Software	FlowJo(v10)
Cell population abundance	Sort-purification was carried out using BC MoFlo Astrios sorter (Beckman),with >98% purity.
Gating strategy	For all experiments, cells of interest were grossly identified by FSC-A/ SSC-A scatter gating, followed by singlet isolation based on FCS-H/FCS-A doublet-exclusion gating. Dead cells were excluded from analysis based on the absence of fixable live/dead viability dye staining, immune cells were then identified by staining of CD45 marker. Detailed gating strategies for specific cell populations were provided in the extended data figures.

☒ Tick this box to confirm that a figure exemplifying the gating strategy is provided in the Supplementary Information.

March 2021
